# ctDNA dynamics demonstrates rapid treatment response to tafasitamab + R-CHOP +/− lenalidomide and predicts outcome in diffuse large B-cell lymphoma: results from the phase 1b First-MIND study

**DOI:** 10.1038/s41375-025-02759-4

**Published:** 2025-10-27

**Authors:** Mouhamad Khouja, Britta Kehden, Derek Blair, Christian Kuffer, Steve Wagner, Tim Versteegen, Philipp Nakov, Monika Brüggemann, Claudia Baldus, David Belada, Grzegorz S. Nowakowski, Anke Schilhabel, Nikos Darzentas, Christiane Pott

**Affiliations:** 1 Second Medical Department, University Hospital Schleswig-Holstein, Kiel, Germany; 2 Clinical Biomarkers & Companion Diagnostics, MorphoSys, a Novartis company Planegg, Planegg, Germany; 3 Clinical Development, MorphoSys, a Novartis company Planegg, Planegg, Germany; 4 4th Department of Internal Medicine - Hematology, Charles University, Hospital and Faculty of Medicine, Hradec Králové, Czech Republic; 5 Division of Hematology, Mayo Clinic, Rochester, MN USA

**Keywords:** Translational research, Genetics research

## Abstract

The firstMIND trial (NCT04134936) evaluated the safety and efficacy of adding lenalidomide to R-CHOP+tafasitamab in the first-line treatment settings of patients with diffuse large B-cell lymphoma. To address response dynamics and the impact of measurable residual disease (MRD), we analyzed prospectively collected plasma samples from 56 study patients using the EuroClonality immunoglobulin gene (IG)-NGS assay. At baseline, disease-related clonotypes were identified in 50/56 (89%) patients by IG-NGS in cell-free (cf)DNA and/or FFPE samples. MRD markers were successfully identified in 49/52 (94%) cfDNA samples and 35/41 (85%) FFPE samples. Baseline cfDNA and circulating tumor (ct)DNA levels correlated with preclinical risk factors, and high cfDNA levels ≥3.35 log_10_hGE/ml plasma were significantly associated with poor progression-free survival (PFS) (hazard ratio (HR): 3.1). ctDNA clearance was rapid with 52% of patients MRD-negative at C2D1, 83% patients at C4D1, and 82% patients after finishing six 21-day cycles (end of treatment (EOT)) and a sustained treatment response (93% MRD negative) six months after EOT. ctDNA detection was associated with worse PFS outcomes at C2D1 (*p* = 0.039, HR:4.51, 95%Cl:0.93–21.74), C4D1 (*p* = 0.07, HR:3.34, 95%Cl:0.83–13.48) and EOT (*p* = 0.01, HR:6.38, 95%Cl:1.27–32.01) and inferior overall survival at these time points. In PET-positive patients, ctDNA-MRD had a higher specificity rendering PET/CT more precisely.

## Introduction

Diffuse Large B-cell Lymphoma (DLBCL) is the most common subtype of non-Hodgkin lymphoma (NHL) accounting for 30–40% of all cases and arising from abnormal B-cells in the lymphatic system [1]. DLBCL is characterized by its rapid growth and aggressive behavior, requiring prompt diagnosis and treatment [2]. The diagnosis of DLBCL is established through a combination of clinical evaluation, histopathological examination, and immunophenotyping of biopsy samples, but mainly relying on a tissue biopsy [2–4]. Moreover, response assessment relies on PET/CT scans at end of therapy, which have imperfect specificity and a moderate positive predictive value of ~50% [5]. Improved staging procedures and molecular follow-up may aid clinicians in guiding treatment decisions and better predicting patient outcomes, enhancing the standard of care for lymphoma patients. In addition, current prognostic factors might be improved to better define patients’ outcomes and help guide treatment decisions. Cell-free (cf)DNA has been validated as a non-invasive analyte for genetic assessment in DLBCL [6–8]. The phase 1b first-MIND trial (NCT04134936) investigated the efficacy of the combination of tafasitamab +/− lenalidomide in combination with R-CHOP in patients with de novo DLBCL, showing a potential benefit of adding lenalidomide to R-CHOP and tafasitamab [9]. Here, we applied the EuroClonality IG-NGS amplicon assay to sensitively identify additional pretreatment risk factors, explore treatment efficacy in real-time, and eventually predict patients at higher risk of relapse.

## Materials and methods

### Patient collection and study design

This analysis was conducted on a series of 56 de novo DLBCL patients treated within the phase Ib trial (firstMIND) by MorphoSys AG (NCT04134936). Patients were randomized into a two-arm study, including six doses of R-CHOP + Tafasitamab as a basis treatment or the addition of Lenalidomide to the basis treatment in an exploratory arm [9–11]. Response assessment was based on PET/CT scans at the end of treatment (EOT). Samples were prospectively collected in cfDNA stabilization tubes (Cell-free DNA BCT, STRECK®) for longitudinal ctDNA testing as defined in the study protocol and ambiently shipped to our lab within 2-5 days.

### Plasma isolation and cfDNA extraction

Blood samples collected in STRECK® tubes were ambiently shipped to our lab for processing. Blood plasma was separated by two centrifugation steps at 1600 x g and 14,000 x g, and stored at −80 °C until further use. cfDNA was purified from up to 4 ml of plasma using the QIAmp Circulating Nucleic Acid Kit (Qiagen, Hilden, Germany) according to the manufacturer’s instructions with extended incubation with Proteinase K to 60 minutes. cfDNA was quantified by three single-plex droplet digital (dd)PCR assays targeting three housekeeping genes [12]. cfDNA levels were reported as the number of haploid genome equivalents (GE) per milliliter of plasma (hGE/mL) and expressed as a log-10 base. cfDNA integrity and purity were assessed on Agilent Bioanalyzer 2100 using the high-sensitivity DNA kit (Agilent Technologies, Germany).

### EuroClonality IG-NGS amplicon library preparation

The academically developed EuroClonality IG-NGS assay was adapted and loci-specific primer sets were modified to suit short cfDNA fragments. A final set of three reactions was used comprising framework region 3 (FR3) of the IGH-VJ gene, as well as IGH-DJ, and IGK rearrangements with a total of 30 forward and five reverse primers (Table S1) [13]. Sequencing libraries were prepared from 100 ng DNA of peripheral blood mononuclear cells (PBMC) or formalin-fixed paraffin-embedded (FFPE) specimens as previously described [14] or from 1500 GE of cfDNA [13] in a one-step PCR (Table S2). For MRD assessment, 5000 GE of cfDNA were used in up to five PCR reactions to reach a sensitivity of 2 × 10^-4^. Forty copies of nine cell lines used in the central in-tube quality/quantification control (cIT-QC) for MRD quantification were added to each reaction to calculate the coverage of each single rearrangement [15]. NGS libraries were sequenced on an Illumina MiSeq (Illumina, Cambridge, UK) using a 2 × 250 bp paired-end chemistry with a final concentration of 7–10 pM according to the manufacturer’s instructions.

### Bioinformatic analysis

Sequencing data was processed by ARResT/Interrogate [16], a standardized bioinformatics platform with wide-ranging immunoprofiling features. The default pipeline was adapted for cfDNA purposes to suit the short fragment size and decrease artifact rates.

### Allele-specific-oligonucleotides (ASO)-based marker validation

Identified IG clonotypes by IG-NGS in cfDNA were validated by allele-specific-oligonucleotide (ASO) ddPCR assay as previously described [17–19]. Briefly, ASO primers specific to the hypervariable N-D-N region of the identified IG clonotype were used with a consensus Jh /Jk reverse primer and a 6-FAM-conjugated primer annealing to a downstream Jh /Jk region for quantification. On average, 2 µl of cfDNA were added to each reaction and amplified according to the PCR conditions in Table S3. Specificity was tested by amplifying polyclonal genomic DNA of pooled healthy donors in each assay.

### Statistical analysis and clinical data interpretation

Progression-free survival (PFS) was defined by time from randomization to stable disease, or progression, whichever occurs first. Overall survival (OS) was defined by time from randomization to death from any cause. PFS and OS were estimated with Kaplan-Meier estimates and compared using log-rank tests. Hazard ratios (HR) were calculated using Cox regression models. Statistical significance between groups for cfDNA and ctDNA levels was estimated using Mann-Whitney tests for unpaired samples. The optimal cut-off for pre-treatment cfDNA levels was determined by repeatedly (2000 times) sampling patients with replacement and, in each iteration, selecting the threshold that best separated patients based on PFS using the log-rank test. All p-values are descriptive only. Data analysis and presentation were performed using R version 4.4.1 (www.R-project.org) using r packages survminer and ggplot2 and GraphPad Prism 8 for Windows (GraphPad Software, USA, www.graphpad.com).

## Results

### Patients’ characteristics

**Fig. 1:**
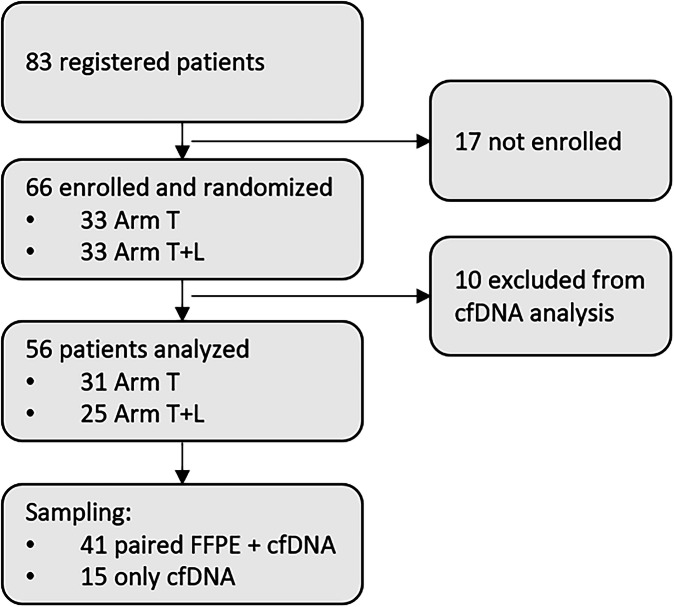
CONSORT Diagram.

A total of 83 patients were initially screened in this trial, 17 screening failures resulted in 66 patients being finally enrolled in the trial. Of these, ten patients had to be excluded from the molecular analysis due to sampling issues, e.g. insufficient sample volume or sampling error detected during analysis (Fig. 1). 25/56 (45%) were men, and the median age was 66.5 years (range: 43–86), 68% were aged >60 years. At baseline, 73% of patients had an Ann Arbor stage IV disease and 44% had a bulky disease. Patients included in the molecular analysis had more frequently a higher international prognostic index (IPI) risk score (4 or 5) in comparison to all enrolled patients (25% vs. 20%, *p* = 0.3). According to Hans-classification for cell-of-origin using immunohistochemistry, 59% of selected patients had germinal-center B-cells (GCB) and 37% were non-GCB (Table 1). Selected patients had comparable PFS and OS outcomes to all enrolled patients with 75% PFS and 95% OS rates at 24-months follow-up (Fig. S1).

**Table 1:** Patient characteristics.

	Complete cohort (n = 66)	Patients with cfDNA analysis (n = 56)
**Age (years)**		
Median (range)	64.5 (20–86)	66.5 (40-86)
**Age categories (years), n (%)**		
≤60	23 (35%)	18 (32%)
>60	43 (65%)	38 (68%)
**Sex, n (%)**		
Male	28 (42%)	25 (45%)
Female	38 (58%)	31 (55%)
**Ann Arbor disease stage, n (%)**		
I	3 (5%)	0
II	1 (2%)	1 (2%)
III	15 (23%)	14 (25%)
IV	47 (71%)	41 (73%)
**IPI risk score, n (%)**		
IPI 2	24 (36%)	17 (30%)
IPI 3	29 (44%)	25 (45%)
IPI 4	11 (17%)	13 (23%)
IPI 5	2 (3%)	1 (2%)
**Elevated LDH levels (≥260 U/L), n (%)**		
Yes	47 (71%)	39 (70%)
No	19 (29%)	17 (30%)
**Bulky disease, n (%)**		
Yes	29 (44%)	25 (44%)
No	37 (56%)	31 (56%)
Cell of origin*, n (%)		
GCB	37 (56%)	33 (59%)
Non-GCB	24 (36%)	21 (37%)
Missing	5 (8%)	2 (4%)

*According to Hans-Classifier.

### cfDNA-based clonality assessment and MRD marker identification

**Fig. 2:**
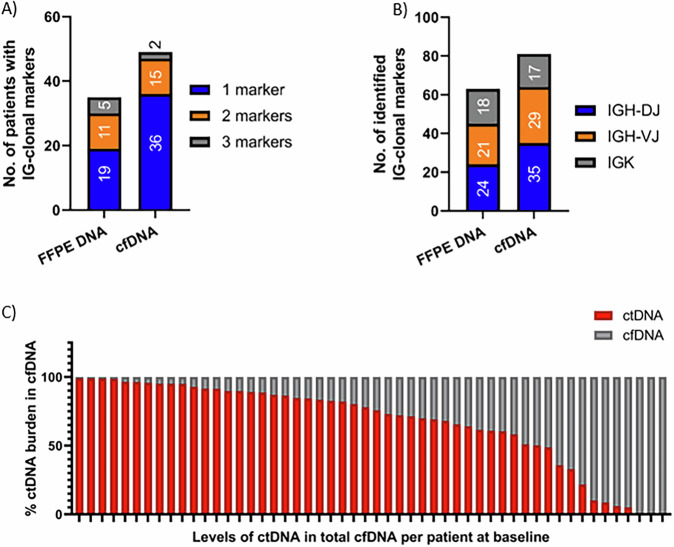
Number of identified IG clonal markers in FFPE and cfDNA. **A** Representation of the number of patients with IG clonal markers in both FFPE DNA and cfDNA according to the number of identified markers per patient. **B** Number of identified IG clonal markers per subtarget in all FFPE DNA or cfDNA samples. **C** Levels of ctDNA in total cfDNA at baseline.

Overall, 337 samples (41 diagnostic FFPE biopsies, 64 PBMC, and 232 plasma samples) from the 56 patients from the Phase Ib First-MIND study in de novo DLBCL (First-MIND; MOR208C107) were analyzed [9]. Paired FFPE, PBMC, and cfDNA samples were available for 41 patients while only PBMC and cfDNA were available for 15 patients. Disease-related clonotypes were identified in 50/56 (89%) patients by IG-NGS in baseline plasma and/or FFPE samples. Baseline cfDNA was informative for IG marker detection in 49/52 (94%) patients (exact 95% Clopper-Pearson confidence interval: 84–99%) with a median clonotype abundance of 11% (range: 1.4–99%) (Fig. 2A, C). IG clonal markers were detected from FFPE samples in 35/41 (85%) patients, one patient had detectable clonotype in FFPE but not in cfDNA. Six patients did not show a clonal marker at all in neither in FFPE nor baseline cfDNA (*n* = 2), or only FFPE (*n* = 4). This was mainly due to polyclonal signals and not to low input DNA/cfDNA amounts.

Among the 49 marker-positive patients from cfDNA, 36 had one IG marker, 11 had two markers and two patients had three suitable IG rearrangements for MRD analysis. Clonal IGH-VJ, IGH-DJ, and IGK rearrangements were detected in cfDNA in 29 (59%), 35 (71%), and 17 (35%) patients, respectively, compared to 21 (60%), 24 (68%), and 18 (51%) in FFPE DNA (Fig. 2B). The IGHV gene usage in cfDNA showed a bias towards IGHV3 (50%), followed by IGHV1 (21%) and IGHV4 (13%) (Fig. S2), however, no association was observed between IGHV gene usage and patient outcomes.

IG clonal markers identified from cfDNA were validated in the 40 paired diagnostic FFPE samples. All IG clonal markers were confirmed in the matched FFPE samples (*n* = 33). In seven FFPE samples, no clonal IG marker was identified by IG-NGS due to technical reasons. The specificity of the IG clonal markers identified by IG-NGS from plasma was further confirmed using ASO ddPCR in 36 cfDNA samples. On average, both methods gave congruent results, however, substantial variability was observed, as indicated by a weak Spearman rank correlation of 0.32 (Figure S3).

### Baseline cfDNA and ctDNA levels correlate with clinical risk parameters and predict patient outcome

**Fig. 3:**
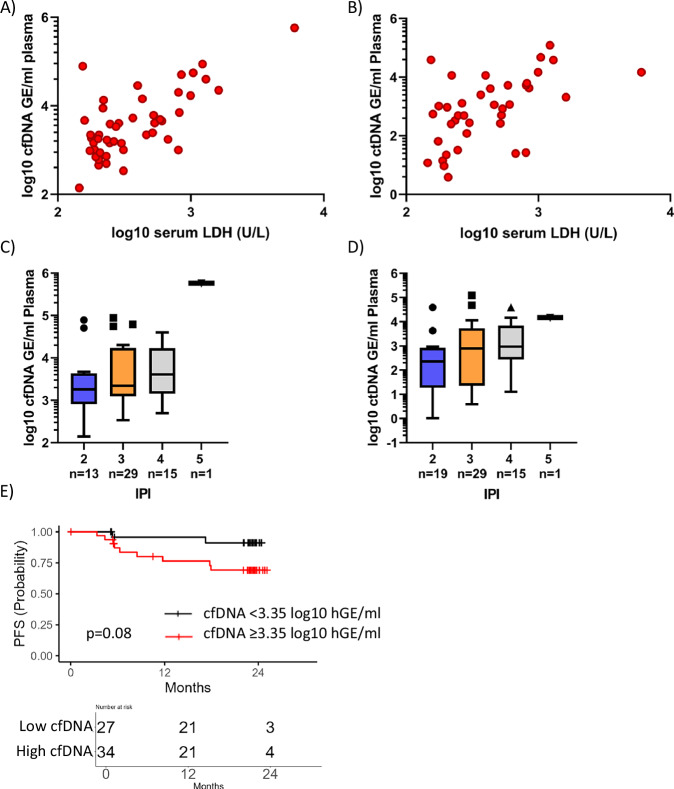
Baseline cfDNA and ctDNA levels in relation to DLBCL clinical risk factors. Relationship between serum lactate dehydrogenase (LDH) levels and pre-treatment cfDNA (**A**) and ctDNA (**B**) concentrations. Association between baseline cfDNA (**C**) and ctDNA (**D**) levels and the international prognostic index. Means are indicated, whiskers indicate to Tukey intervals. (**E)** Kaplan-Meier plots showing progression-free survival stratified by cfDNA levels ≥3.35 log10hGE/ml at time of diagnosis.

Total baseline cfDNA and ctDNA levels correlated with pre-treatment serum lactate dehydrogenase (LDH) levels and IPI scores (Fig. 3A–D). Patients with elevated cfDNA (≥3.35 log_10_hGE/ml, as defined by a Cox regression model) showed a trend towards inferior PFS (*p* = 0.13, HR = 3.1, 95% Cl=0.64–14.95) (Fig. 3E). Median baseline ctDNA levels were comparable between treatment arms (2.70 and 2.71 log_10_hGE/ml).

### ctDNA dynamics demonstrate treatment response and predict patient outcome

**Fig. 4:**
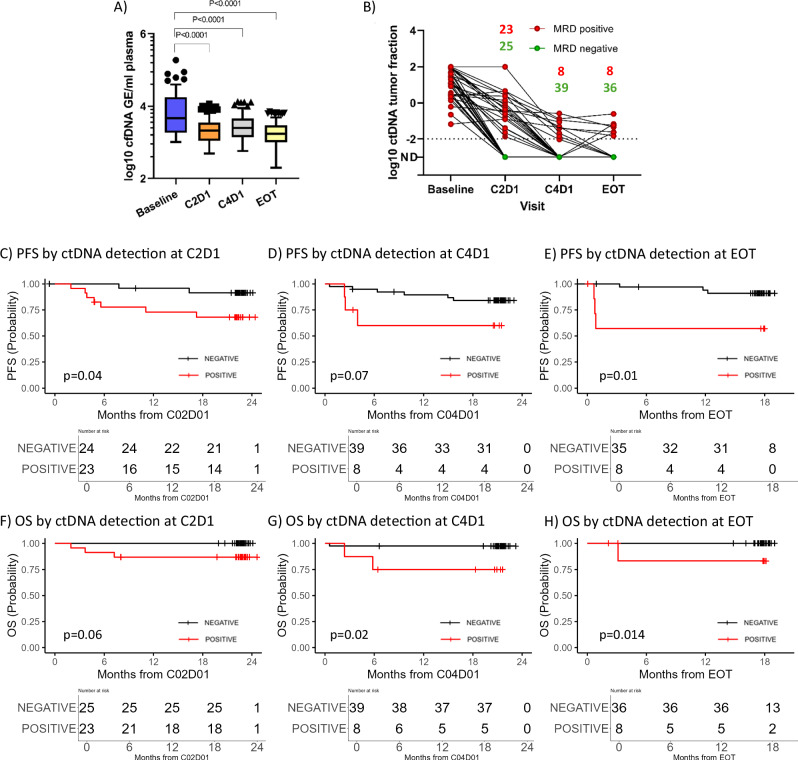
ctDNA dynamics demonstrate treatment response and predict patient outcome. **A** Levels of total cfDNA per mL of plasma during induction therapy. Means are indicated, whiskers indicate Tukey intervals. Significance is estimated by one-way ANOVA. **B** Dynamics of ctDNA detected during and after treatment, the number of patients with detectable ctDNA are shown in red while green indicates to undetectable ctDNA. **C**–**E** Progression-free survival rates stratified by ctDNA detection at C2D1, C4D1, and EOT, respectively. **F**–**H** Overall survival rates stratified by ctDNA detection at C2D1, C4D1, and EOT, respectively. Black curves indicate to ctDNA negativity and red curves indicate to ctDNA positivity.

Total cfDNA levels were significantly decreased after the first treatment cycle (*p* = 0.0001) indicating an early and rapid cell turnover within the first treatment cycle (Fig. 4A). Moreover, dynamics of ctDNA detected during and after treatment show effective and rapid ctDNA clearance in 25/48 (52%) patients at cycle 2 day 1 (C2D1), 39/47 (83%) patients at C4D1 and 36/44 (82%) patients at EOT. ctDNA clearance appeared in 38/41 (93%) at six months follow-up (FU) after EOT (Fig. 4B). Patients receiving lenalidomide in the experimental arm (*n* = 30) showed a trend towards better disease clearance than patients in the control arm represented by a higher number of ctDNA-negative patients at C2D1 (57 vs. 52%), which was consistent at C4D1 (87 vs. 79%). At EOT, levels of ctDNA negativity were comparable in both treatment arms (82 vs. 81%). Patients with non-GCB DLBCL had slightly better ctDNA clearance at C2D1 than patient with GCB DLBCL (53 vs. 48%), but not at later timepoints (C4D1 and EOT), where ctDNA negativity rates were comparable among COO subtypes. ctDNA detection, reflecting residual disease, predicted inferior PFS at C2D1 (*p* = 0.039, HR: 4.51, 95% Cl: 0.93–21.74, 24-months PFS: 68 vs. 91%), C4D1 (*p* = 0.07, HR: 3.34, 95% Cl: 0.83–13.48, 18-months PFS: 60 vs. 84%) and EOT (*p* = 0.01, HR: 6.38, 95% Cl: 1.27–32.01, 18-months PFS: 60 vs. 91%) (Fig. 4C–E). Inferior OS rates were associated with ctDNA detection at C2D1 (*p* = 0.06, HR: too high to be defined, 95% CI: 0-Inf, 18-months OS: 87 vs. 100%), at C4D1 (*p* = 0.02, HR: 10, 95% CI: 0.91–110, 18-months OS: 75 vs. 97%) and at EOT (*p* = 0.014, HR: too high to be defined, 95% CI: 0-Inf, 18-months OS: 87 vs. 100%) (Fig. 4F–H).

The overall response at EOT of the cohort analyzed in this paper was 78.4%, eight patients were PET-positive (five with partial remission and three with progressive disease). Interestingly, ctDNA negativity (*n* = 23) or reduction to <1% (*n* = 12) at C2D1 predicted complete response assessed by PET/CT at EOT. Moreover, ctDNA measurement at EOT in the eight PET-positive patients showed only three ctDNA-positive cases while five were negative. None of the five ctDNA-negative patients, classified as PET PR, relapsed at a median observation time of 24 months, but all ctDNA-positive PET-positive patients relapsed within the first six months. ctDNA measurement at EOT identified false PET positivity, as shown in Fig. S4 and seems to be more specific for the prediction of relapse.

## Discussion

The Phase 1b First-MIND trial investigated the feasibility of adding tafasitamab ± lenalidomide to R-CHOP regimen in the first-line setting in patients with DLBCL, demonstrating a manageable safety profile and a promising efficacy [9]. To gain insight into response kinetics and to assess treatment efficacy with a highly sensitive method, we studied ctDNA using the EuroClonality IG-NGS assay at predefined time points during and after treatment in this uniformly treated study cohort. Our results show that clonotype identification for MRD assessment can be reliably performed from baseline cfDNA in 84% of patients using our IG-NGS assay, however, detection of IGK clonotypes in cfDNA was less informative than in FFPE DNA, this might be due to the larger amplicon size of 300 bp in IGK than ~250 bp in complete and incomplete IGH clonotypes. The ability to perform tumor-uninformed marker detection from liquid biopsy provides a major advantage for the applicability of MRD within and outside of clinical trials, circumventing the need for tumor biopsies and easing the burden on patients. Our results confirm the feasibility of cfDNA as a diagnostic specimen in patients with DLBCL. When comparing IG-NGS and ddPCR for ctDNA quantification in baseline plasma samples, quantitative ctDNA levels substantially varied between the two methods and between the different targets. Non-PCR-based procedures, such as targeted-capture based sequencing (CAPP-Seq) and Phased-variant enrichment and sequencing, which focus on multiple variants as MRD targets rather than a single rearrangement for MRD tracking, might provide less variable MRD quantification. Pretreatment ctDNA has been shown to be a surrogate for total metabolic tumor volume and to predict outcome as an independent variable [7, 20–22]. We investigated the relationship between cfDNA and ctDNA quantitative levels at diagnosis and known clinical prognostic markers such as IPI and LDH and confirmed the prognostic value of pretreatment cfDNA and ctDNA level, confirming its role as a prognostic biomarker in DLBCL. We observed a predictive value of baseline cfDNA levels (≥3.35 log_10_hGE/ml) for inferior outcomes determined by an univariable Cox regression. This threshold defining high pretreatment cfDNA levels is higher than the threshold previously published by Kurtz et al., namely 2.5 log_10_hGE/ml [23], because the latter did not adequately split our cohort. On the one hand, this could be due to the fact that our cohort was enriched for patients with IPI scores 3–5 (80 vs. 40%) compared to the previously published series, but could also reflect differences in methods applied for ctDNA quantification. In the study of Kurtz, cancer personalized profiling by deep sequencing (CAPP-Seq) by mutational genotyping was used for ctDNA assessment, while our approach is based on the quantification of clonal immunoglobulin rearrangements through high-throughput sequencing. As IG-NGS is a robust NGS method with a low technical background error profile for ctDNA quantification, one would like to establish method-agnostic thresholds for ctDNA quantification to allow a universal (and cost-efficient) application of pretreatment ctDNA quantitative levels to refine staging with the focus on precise risk factors. Further studies are needed to compare technical applicability, define sensitivity thresholds, and standardize its use.

The EuroClonality IG-NGS assay is well suited for dynamic response assessment in cfDNA samples during and after treatment and confirms previously published results demonstrating superior outcomes associated with ctDNA clearance early during treatment. In particular, ctDNA assessment showed early and rapid molecular disease clearance after only one treatment cycle of tafasitamab ± lenalidomide and R CHOP, identifying at this early time point those patients who will achieve a PET-CT response after EOT and have a subsequent good prognosis. Early ctDNA clearance was associated with superior PFS and OS outcomes in comparison to patients with detectable ctDNA at EOT. This is in accordance with results published from a phase 2 study of DA-EPOCH-R or R-CHOP +/− acalabrutinib for treatment naïve DLBCL by Roscheswki et al. [24], however, patients with detectable ctDNA had worse 18-months PFS of ~30% PFS compared to 60% PFS in our study. This might in part be attributed to methodical differences in a small patient cohort but also to the fact that one of the three EOT MRD-negative patients relapsing between 10 and 24 months after treatment had an extranodal CNS relapse that could not be detected in plasma. For clinical trials testing early interventional strategies, according to our data, ctDNA status at C2D1 might be a useful time point for individual risk-based intervention strategies. Our findings align with previous reports indicating that early and deep ctDNA reduction after one treatment cycle is prognostic for patient outcomes [7, 23]. Interestingly, we were also able to show the additive effect of lenalidomide added to the combination of tafasitamab+R-CHOP. We observed a faster ctDNA clearance at early induction time points in patients receiving lenalidomide and tafasitamab+R-CHOP, while this effect was obviously compensated by additional treatment cycles at EOT. This will allow in the future, instead of performing multiple PET scans with implied radiation exposure, to evaluate specific treatment elements and their impact on treatment efficiency and outcome by a single ctDNA analysis. Further analysis of ctDNA at later follow-up timepoints and investigating disease kinetics in the front-MIND phase III trial might better define the value of lenalidomide addition to tafasitamab+R-CHOP in the first-line setting.

Our IG-NGS MRD results were highly predictive for outcomes, although IG-NGS-based MRD detection is known to be less sensitive than approaches like CAPP-Seq using mutational genotyping [25]. This might be due to our strategy to analyze a minimum amount of 5000 GE per MRD reaction instead of variable amounts of input DNA according to a fixed plasma volume per reaction.

Until recently, PET/CT imaging after EOT was the standard approach for assessing response after induction therapy in aggressive B-cell lymphomas. While PET/CT provides valuable prognostic classification, its sensitivity and specificity have known limitations. In this context, ctDNA analysis has emerged as a promising alternative for response monitoring, increasing the specificity of response assessment [24]. We analyzed PET-positive patients according to their ctDNA status at EOT and detected false PET-positivity in 5/8 cases. Patients with positive PET/CTs and detectable ctDNA at EOT (*n* = 3) progressed within 6 months, whereas ctDNA-negative patients at EOT (*n* = 5) remained in remission. Although this is a small number of patients, our results with a less sensitive assay discriminate between active malignancy and unspecific lesions in PET-positive patients and confirm the previous report by Roschewski et al. [24]. This group showed a landmark comparison of a singular PET/CT Scan after EOT versus ultrasensitive ctDNA detection with an analytical detection threshold (~1:10^6^ cfDNA molecules) and showed higher specificity and positive predictive value of EOT MRD assessment in cfDNA [24]. The importance of ctDNA as a sensitive and specific tool for response assessment has been addressed by the recent recommendations of the National Comprehensive Cancer Network (NCCN) Clinical Practice Guidelines in Oncology for B-cell lymphoma, which include ctDNA measurements as an alternative to biopsy for the evaluation of positive PET imaging results for patients who have achieved PET partial response or progressive disease at the end of frontline DLBCL therapy.

Several approaches for ctDNA quantification are currently available and ctDNA assessment will eventually be established as a complementary biomarker. The results of this study highlight the utility of an academic IG-NGS-based ctDNA MRD assay that is robust and sensitive for ctDNA detection and measurement in aggressive B-cell lymphomas. In addition to providing accurate response assessment, it allows dynamic monitoring throughout the treatment course in patients with DLBCL, providing insight into disease kinetics and windows for treatment intervention in clinical trials. It also allows the impact assessment of different therapeutic regimens.

## Supplementary information

Supplemental figures

Supplementary tables

## Data Availability

The raw sequencing data analyzed in this study is available from the corresponding author on a reasonable request.
