# Supplementary material for: ctDNA dynamics demonstrates rapid treatment response to tafasitamab + R-CHOP +/− lenalidomide and predicts outcome in diffuse large B-cell lymphoma: results from the phase 1b First-MIND study
Source: Leukemia. 2025 Oct 27;40(1):87–94. doi: 10.1038/s41375-025-02759-4 (PMC12789008; doi:10.1038/s41375-025-02759-4)
Supplement: Supplementary file 1 — Supplemental figures [file 41375_2025_2759_MOESM1_ESM.docx]

**Supplementary Material**

**ctDNA dynamics demonstrates rapid treatment response to tafasitamab + R-CHOP +/- lenalidomide and predicts outcome in diffuse large B-cell lymphoma: results from the phase 1b First-MIND study**

Mouhamad Khouja^1^, Britta Kehden^1^, Derek Blair^2^, Christian Kuffer^2^, Steve Wagner^3^, Tim Versteegen^1^, Philipp Nakov^1^, Monika Brüggemann^1^, Claudia Baldus^1^, David Belada^4^, Grzegorz S Nowakowski^5^, Anke Schilhabel^1^, Nikos Darzentas^1^, Christiane Pott^1^ on behalf of the EuroClonality-NGS Working Group.

Affiliations:

1 Second Medical Department, University Hospital Schleswig-Holstein, Kiel, Germany

2 Clinical Biomarkers & Companion Diagnostics, MorphoSys, a Novartis company Planegg, Germany

3 Clinical Development, MorphoSys, a Novartis company Planegg, Germany.

4 4^th^ Department of Internal Medicine - Hematology, Charles University, Hospital and Faculty of Medicine, Hradec Králové, Czech Republic

5 Division of Hematology, Mayo Clinic, Rochester, MN

Corresponding author

Prof. Dr. med. Christiane Pott

Second Medical Department

University Hospital Schleswig-Holstein, Campus Kiel

Arnold-Heller Str. 3

24105 Kiel

Germany

Phone: ++49-431-500-24981

Fax: ++49-431-500-22554

Email: [c.pott@med2.uni-kiel.de](mailto:c.pott@med2.uni-kiel.de)

**Figure S1** Progression-free and overall survival rates of the entire cohort.


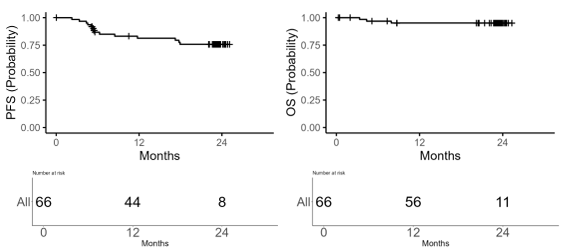


**Figure S2** Circular representation of IGHV-D-J usage in the analyzed cohort showing a bias towards IGHV3, IGHV1, and IGHV4.

**
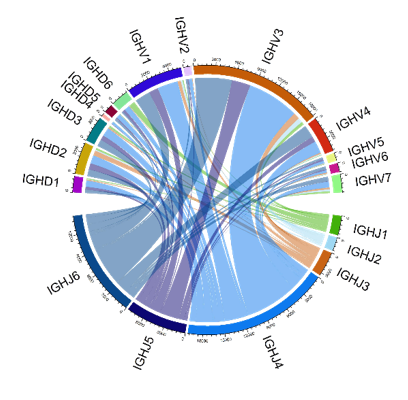
**

**Figure S3** Scatterplot ASO ddPCR versus IG-NGS. Red, green and blue points indicate results for IGH-DJ (n=21), IGH-VJ (n=17) and IGK (n=10), respectively. Black line on grey shading indicate the Passing-Bablok regression line with the 95% confidence band. Axes are on decimal logarithmic scale.

**
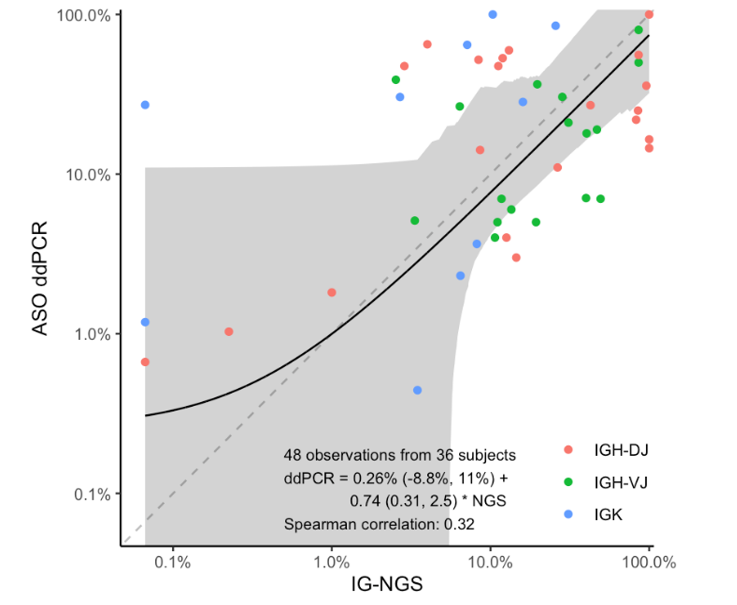
**

**Figure S4** Predictive value of ctDNA detection at end of treatment in PET-positive patients. Black curves indicate ctDNA negativity and red curves indicate ctDNA positivity.

**
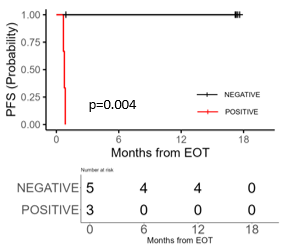
**
